# LMF-CP: An Interpretable Multimodal Late-Fusion Framework for Compound Carcinogenicity Prediction

**DOI:** 10.3390/ijms27167499

**Published:** 2026-08-21

**Authors:** Yingjie Zhu, Liujie He, Xinjie Liang

**Affiliations:** School of Mathematics and Statistics, Changchun University, Changchun 130022, China; 19591370365@163.com (L.H.); 135lxjyyds@gmail.com (X.L.)

**Keywords:** carcinogenicity, multimodal learning, deep learning, interpretable analysis, feature fusion

## Abstract

Accurately predicting the carcinogenicity of compounds is of great significance for drug discovery, clinical drug safety, and chemical risk assessment. Traditional methods for assessing carcinogenicity rely on animal testing, which suffers from limitations such as time-consuming processes, high costs, significant interspecies differences, and low predictive throughput. In recent years, computational modeling-based prediction methods (such as Quantitative Structure–Activity Relationships, QSAR) have made some progress, but they still face challenges such as insufficient molecular feature information and poor model interpretability. To overcome these barriers, the multimodal deep learning framework LMF-CP (Late Multimodal Fusion of Carcinogenicity Prediction) is proposed to enhance the performance and interpretability of compound carcinogenicity prediction. First, to comprehensively characterize the structural and physicochemical properties of compounds, a multimodal representation system based on four molecular modalities is constructed, namely SMILES sequences, molecular fingerprints, molecular images, and molecular graph structures. Specifically, Text Convolutional Neural Network (TextCNN), Multi-Layer Perceptron (MLP), Visual Geometry Group Network (VGGNet), as well as Molecular Graph Attention Network (MGAT) are employed to process this information, respectively. Second, to integrate information from different molecular representations, a late-stage fusion strategy based on Lasso stacking is employed. On the test set, LMF-CP achieves an area under curve (AUC) of 0.828, an accuracy (ACC) of 0.782, an F1 score of 0.786, a sensitivity (SEN) of 0.786, and a specificity (SPE) of 0.779. In addition, this paper combines Shapley Additive Explanations (SHAP) analysis with Bemis–Murcko scaffold analysis to interpret the model results from two perspectives. Finally, a visual online platform for predicting the carcinogenicity of compounds is designed, providing a convenient tool for the rapid assessment of compound carcinogenicity and structural interpretation.

## 1. Introduction

Cancer is one of the leading causes of death worldwide. According to statistics, there were 20.6 million new cases and more than 9.8 million deaths from cancer in 2024 [1]. In terms of cancer incidence, breast cancer, lung cancer, colorectal cancer, and prostate cancer are the four most common types of cancer [2]. There are many factors that contribute to cancer, including environmental, genetic, and psychological factors, among which environmental factors account for the majority of cancer causes [3]. Environmental factors encompass lifestyle, diet, smoking, and pollution, of which chemical carcinogens are a significant contributing factor [4]. Studies have shown that environmental factors, particularly chemicals, account for 70–90% of cancer cases [5]. Therefore, these chemical carcinogens require close attention.

In the field of drug discovery and development, carcinogenicity is also one of the key factors influencing drug safety assessments. The potential carcinogenic risk of the drug not only affects the safety of its clinical application but also directly impacts its development process and marketing approval [6,7]. According to relevant regulatory requirements, systematic carcinogenicity evaluation studies are typically required before a new drug is approved for marketing. At the current stage of research, our understanding of carcinogens relies primarily on data generated from carcinogenicity testing in rodents. Relevant studies indicate that most chemicals associated with human carcinogenicity induce tumors in rats and mice [8]. However, traditional animal testing methods face numerous challenges. First, experiments are time-consuming and costly, as a two-year carcinogenicity study typically requires over a hundred laboratory animals and can cost up to millions of dollars [3]. Second, there are certain differences in the correlation between experimental results and human carcinogenic risks [9]. Finally, traditional animal testing struggles to assess carcinogenicity with large numbers of chemicals on a one-to-one basis [10]. Therefore, it is urgently necessary to develop an efficient, low-cost, high-throughput method for screening carcinogens.

In recent years, an increasing number of methods have been utilized to predict the carcinogenicity of compounds. These can be primarily classified into three categories comprising expert systems, Structure–Activity Relationship (SAR) models, and Quantitative Structure–Activity Relationship (QSAR) models [11,12,13]. Expert rule models are a crucial component of computer-aided tools [14,15]. The SAR and QSAR models establish qualitative and quantitative relationships between molecular features and chemical carcinogenicity [3]. Early studies relied largely on manually selected molecular descriptors, often combined with traditional machine learning algorithms. For example, researchers used Counter-Propagation Artificial Neural Network (CP-ANN) for classification [16] or multiple regression analysis to predict the TD50 of carcinogens [17]. To improve predictive accuracy, researchers incorporated support vector machines (SVM) and variable selection techniques, significantly enhancing the model’s predictive performance through optimized feature selection [18,19]. In addition, the ensemble models (such as CarcinoPred-EL) [20] achieved more stable carcinogenicity predictions by integrating seven molecular fingerprints with an average accuracy of 70% on the test set. Finally, other methods (such as Naive Bayes models and Random Forest models) were also widely used for carcinogenicity prediction [21,22]. Although the aforementioned calculation methods have achieved reasonable predictive capabilities, they still have some limitations in distinguishing carcinogenic and non-carcinogenic substances.

With the advancement of artificial intelligence technology, deep learning has been widely applied in computer-aided drug design. In 2022, Fradkin et al. [23] were the first to use graph neural networks and employ a transfer learning model to identify carcinogenic molecules, achieving good performance. In the same year, a hybrid neural network model was proposed, which improved the generalization ability of carcinogens [24]. Subsequently, classification models based on capsule networks and attention mechanisms were applied to carcinogenicity prediction, enabling more refined feature extraction and information fusion [25]. In 2025, significant progress was made in multimodal deep learning methods [26]. A multimodal deep model was proposed that integrates molecular graphs, molecular fingerprints, and two-dimensional molecular images to significantly improve model performance. In addition, another study developed a multi-model consensus framework to conduct a robust assessment of potential carcinogens of the International Agency for Research on Cancer (IARC) Group 2B, achieving both predictive robustness and interpretability [27]. These results demonstrate that deep learning models can enhance predictive accuracy and generalization capabilities while also offering new directions for model interpretability and practical applications.

Although significant progress has been made in recent years in developing carcinogenicity classification and dose prediction models based on machine learning and deep learning, existing models still have common limitations. On the one hand, most current studies still rely on single-modality data for modeling and make it difficult to fully characterize the multidimensional information of molecules, which limits the predictive capabilities of these models. To overcome this limitation, multimodal learning has gradually emerged as a hot research topic, which has shown significant potential in fields such as drug design [28]. However, there may be redundancy, complementarity, or synergy among different modalities [29,30], and how to effectively integrate multimodal information remains a key challenge. Existing multimodal techniques include early fusion, late fusion, hybrid fusion, and model ensemble methods [31,32,33,34,35,36,37]. Therefore, the development of learning methods capable of optimizing the fusion of multimodal information will significantly advance the precision and widespread application of carcinogenicity prediction. On the other hand, interpretability is one of the major challenges facing deep learning models [34]. Although current predictive methods have improved in terms of accuracy, they often lack transparency and intuitiveness, making it difficult for researchers to understand the basis for model decisions.

To address the above issues, a framework based on late multimodal fusion for predicting molecular carcinogenicity called LMF-CP (Late Multimodal Fusion of Carcinogenicity Prediction) is proposed. This framework is of significant importance for improving the accuracy of existing computational models and enhancing the interpretability of results. The main contributions of this study are summarized as follows.

- By integrating Simplified Molecular Input Line Entry System (SMILES) sequences, molecular fingerprints, molecular graphs, and molecular images, we have developed the LMF-CP framework. This multimodal molecular carcinogenicity prediction model enables a comprehensive characterization of molecular features from multiple perspectives.
- To achieve effective integration of multimodal information, a late-fusion strategy based on Lasso stacking is employed. The prediction outputs of individual modalities are combined at the decision level using a $L_{1}$-regularized logistic regression meta-learner. We also evaluate three alternative fusion strategies, namely simple concatenation, attention-weighted fusion, and stacking based on stochastic gradient descent (SGD).
- The project has achieved both an interpretability analysis and practical application of the results. In terms of interpretability, Shapley Additive Explanations (SHAP) analysis and Bemis-Murcko scaffold analysis are combined to identify prediction-associated substructural patterns and complementary scaffold-level characteristics. In terms of application, an online prediction platform is developed to generate prediction results, three-dimensional (3D) molecular structures, and physicochemical properties, thereby establishing an integrated “prediction–interpretation–properties” workflow. This provides researchers with a convenient analytical tool and enhances the practical value of the model.

## 2. Results

### 2.1. Performance Evaluation

To evaluate the classification performance of the established model, five-fold cross-validation is employed for assessment. Specifically, four of the five folds are utilized as training data, while the remaining one serves as validation data to test the model. The following metrics are calculated: Area Under the Curve (AUC), Accuracy (ACC), Sensitivity (SEN), Specificity (SPE), and F1 Score. The calculation formulas for these metrics are as follows:

(1) $$\mathrm{SEN}=\frac{\mathrm{TP}}{\mathrm{TP}+\mathrm{FN}},$$

(2) $$\mathrm{SPE}=\frac{\mathrm{TN}}{\mathrm{TN}+\mathrm{FP}},$$

(3) $$\mathrm{ACC}=\frac{\mathrm{TP}+\mathrm{TN}}{\mathrm{TP}+\mathrm{TN}+\mathrm{FN}+\mathrm{FP}},$$

(4) $$F1\mathrm{score}=\frac{2\mathrm{TP}}{2\mathrm{TP}+\mathrm{FP}+\mathrm{FN}}.$$

TP (True Positives) represents the number of correctly predicted “carcinogens”; TN (True Negatives) represents the number of correctly predicted “non-carcinogens”; FP (False Positives) represents the number of “non-carcinogens” incorrectly predicted as “carcinogens”; and FN (False Negatives) represents the number of “carcinogens” incorrectly predicted as “non-carcinogens”. AUC represents the area under the ROC curve, serving as a crucial metric for classifiers, and its value ranges between 0 and 1, with magnitude directly indicating the predictive performance of the model.

### 2.2. Multimodal Feature Space Distribution Visualization

**Figure 1:**
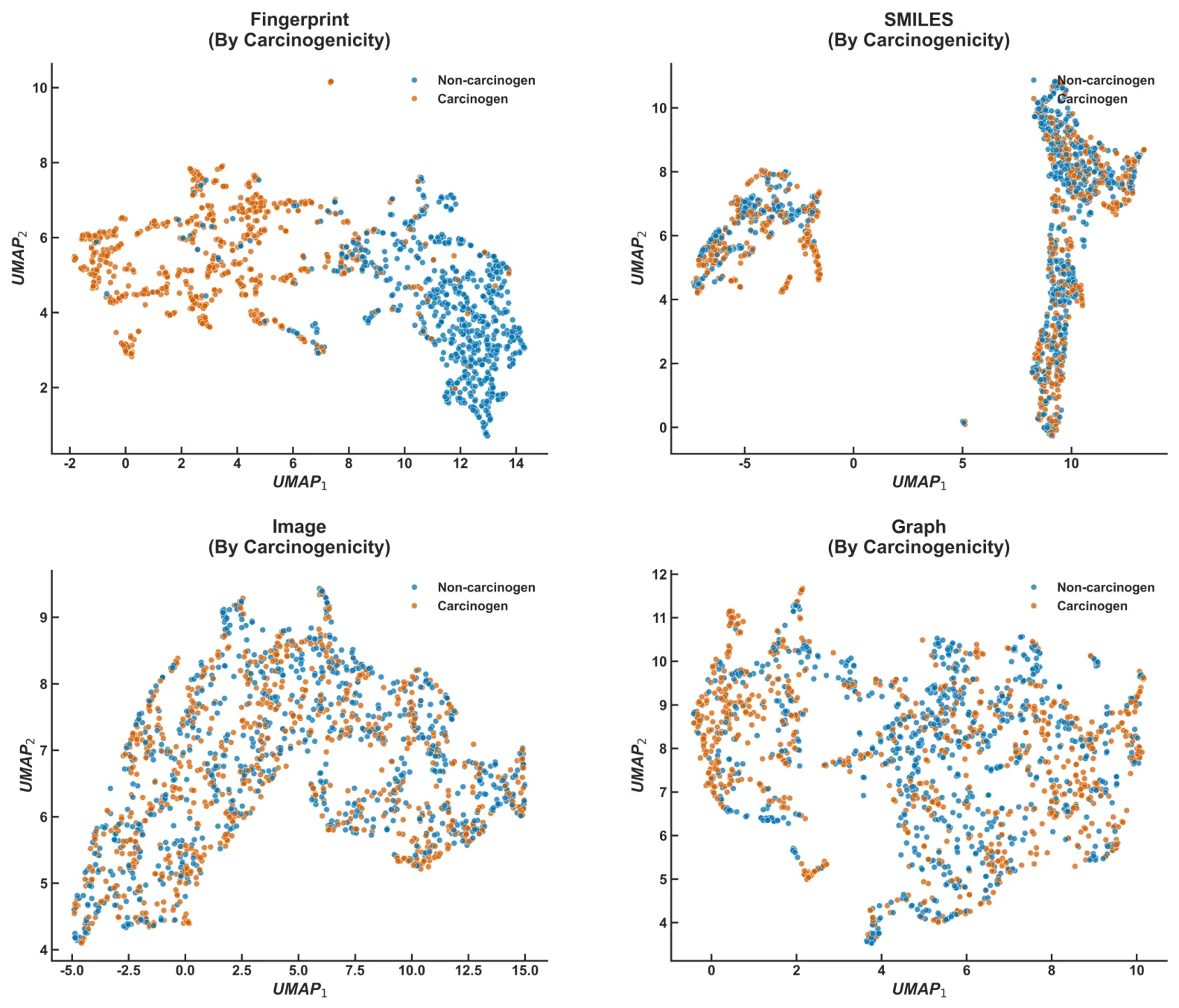
Uniform Manifold Approximation and Projection (UMAP) dimensionality reduction diagram. The figure is colored according to compound carcinogenicity (orange for carcinogens and blue for non-carcinogens).

To study the relationships among different input features, a dimension reduction method called Uniform Manifold Approximation and Projection (UMAP) is used to visualize the spatial distribution of these features. By combining the results shown in Figure 1, we can observe that when one molecular representation method results in only slight overlap between data points, another representation method may lead to significant overlap [38]. Among these, molecular fingerprints exhibit a relatively clear separation trend, while the feature spaces of SMILES, molecular graphs, and images show overlapping distributions. However, molecular fingerprints, such as Molecular ACCess System (MACCS) fingerprints rely on predefined structural fragments, resulting in blind spots to identify unknown toxicity-alert structures. SMILES sequence features focus on contextual information, molecular graphs emphasize topological structures and interatomic relationships, and images focus on visual geometric features. Each of these representation methods has its own strengths; thus, a single modality struggles to comprehensively characterize the complex chemical properties of compounds.

Based on the analysis above, this paper proposes a multimodal fusion strategy to construct a more comprehensive chemical space representation.

### 2.3. Comparison of Multimodal Fusion Strategies

To determine the most suitable multimodal fusion strategy for LMF-CP, this study compares four methods, including simple concatenation, attention-weighted fusion, SGD stacking, and Lasso stacking. We use the average AUC from fivefold cross-validation as the primary criterion for selecting the fusion strategy, and evaluate their overall performance by combining ACC, AUC, SEN, and SPE. The results for different fusion strategies are shown in Table 1. The results indicate that the Lasso stacking method achieved the highest mean AUC of 0.8405 and also outperformed the other three fusion strategies in terms of mean ACC.

Additionally, the results of the Wilcoxon test [39] (Supplementary Table S1) show that the differences in performance between Lasso stacking and the other three strategies are not statistically significant (p=0.625, p=0.438 and p=0.313). Although the differences are not statistically significant, Lasso stacking achieves the highest mean cross-validation AUC, which is defined as the primary criterion for fusion-strategy selection. Therefore, Lasso stacking is selected as the final fusion strategy.

### 2.4. Comparison with Other Models

To evaluate the performance of the proposed LMF-CP model in compound carcinogenicity prediction, we compare it with three classical algorithms, namely K-nearest neighbors (KNN) [40], SVM [41], and graph convolutional networks (GCNs) [23]. In addition, DCAMCP is a molecular carcinogenicity prediction model based on capsule networks that combines molecular fingerprint features and molecular graph structures to represent molecular information and uses capsule networks to model the hierarchical relationships among features; thus, its ability to characterize complex molecular structures is enhanced [25]. The corresponding results are shown in Table 2.

As shown in Table 2, LMF-CP performs best across most evaluation metrics, with average values of 0.7738 for ACC, 0.8405 for AUC, 0.7896 for SEN, and 0.7576 for SPE. Compared to traditional machine learning models, LMF-CP obtains higher mean AUC values. It can be seen that the AUC of LMF-CP is 0.8405, which is higher than KNN, SVM, and GCN, achieving improvements of 10.65%, 6.34%, and 7.11%, respectively. These results demonstrate that LMF-CP achieves favorable overall predictive performance over traditional machine learning methods and standard graph neural networks. Then, compared to the relatively advanced baseline model DCAMCP, LMF-CP achieves a 7.17% improvement in ACC, AUC by 4.33%, and SPE by 23.10%. Although DCAMCP achieves the highest sensitivity value of 0.8260, its SPE is low at only 0.6154. In general, LMF-CP achieves the highest mean AUC and ACC among the models included in Table 2.

Subsequently, statistical analysis of the paired results from the five-fold cross-validation is performed. In terms of AUC, ACC, and F1 scores, LMF-CP yields higher values than KNN, SVM, GCN, and DCAMCP across all five folds (Supplementary Table S2). Although the Wilcoxon *p*-value does not reach the conventional significance level due to the limited statistical power of the fivefold cross-validation, the consistent superiority of LMF-CP across all folds and the maximum effect size (rank-biserial = 1.000) provide a reference point for the relative stability of the model’s performance.

To further expand the comparison with pretrained molecular representation methods developed in recent years, this study additionally introduces two representative pretrained models (Mole-BERT and ChemBERTa-2) [42,43]. LMF-CP achieves an average AUC of 0.8405, which is higher than the 0.7669 achieved by Mole-BERT and the 0.7408 achieved by ChemBERTa-2, indicating that the proposed multimodal framework still demonstrates good overall predictive performance when compared with pretrained molecular representation methods (Supplementary Table S3).

Additionally, a randomized-label negative control experiment is conducted to assess whether the predictive performance of LMF-CP can arise from random label patterns. The model performance decreases markedly after label permutation, with the overall AUC and balanced accuracy approaching the chance level of 0.5 (Supplementary Table S4). This result supports the idea that the predictive performance of LMF-CP depends on meaningful associations between molecular representations and carcinogenicity labels rather than random label patterns.

### 2.5. Evaluation on the Test Set

**Figure 2:**
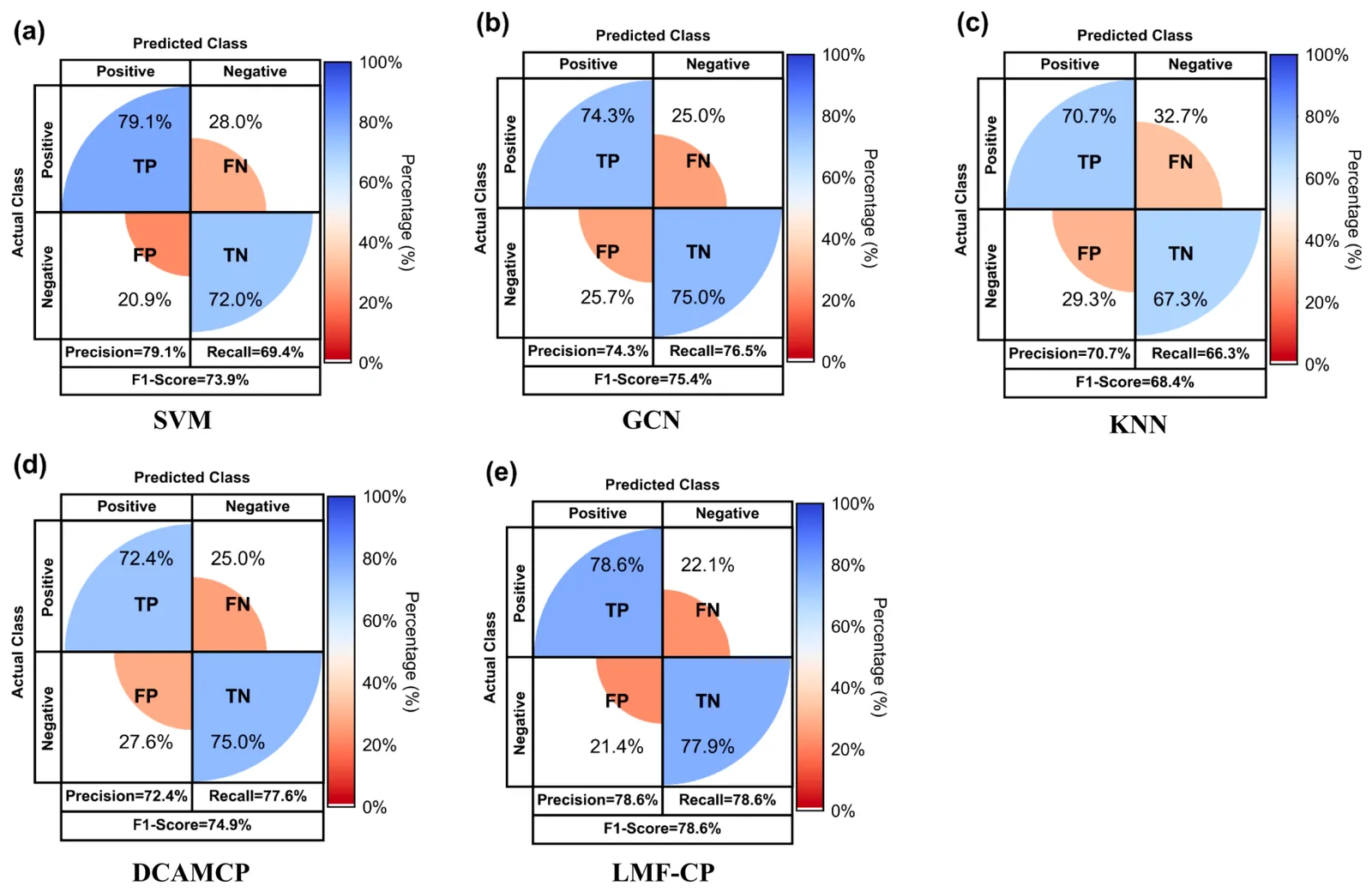
Confusion matrix visualization on the test set: (**a**) SVM; (**b**) GCN; (**c**) KNN; (**d**) DCAMCP; and (**e**) LMF-CP. Each subgraph contains four sector areas representing True Positive (TP), False Positive (FP), False Negative (FN), and True Negative (TN). The vertical axis represents the actual class, while the horizontal axis denotes the predicted class. Below each subgraph, three key performance metrics are displayed: Precision, Recall, and F1-Score.

This section evaluates the predictive performance of LMF-CP on the test set that was not used during model training or parameter tuning. Therefore, the test set provides an additional assessment of the model’s predictive performance on unseen compounds within the current dataset. The specific experimental results are shown in Table 3, and the corresponding confusion matrices are presented in Figure 2.

As shown in Table 3 and Figure 2, LMF-CP achieves an AUC of 0.828, an ACC of 0.782, a SEN of 0.786, a SPE of 0.779, and an F1 score of 0.786 on the test set. Among the evaluated models, LMF-CP achieves the highest AUC, ACC, SEN, and F1 score, whereas SVM achieves the highest SPE. Compared with KNN, LMF-CP shows relative improvements of 9.81%, 13.5%, 14.91%, 18.55%, and 8.95% in AUC, ACC, F1, SEN, and SPE, respectively. Compared with GCN, the corresponding relative improvements are 4.55%, 4.83%, 4.24%, 2.75%, and 7.3%, respectively. Compared with DCAMCP, LMF-CP shows relative improvements of 2.48%, 6.39%, 4.94%, 1.42%, and 12.25% in AUC, ACC, F1, SEN, and SPE, respectively. Compared with SVM, LMF-CP shows relative improvements of 1.35%, 4.13%, 6.36%, and 13.26% in AUC, ACC, F1, and SEN, respectively, while SPE decreases by 3.83%. However, its comparative advantages are not uniform across all evaluation metrics and baseline models, indicating that improvements in sensitivity may be accompanied by a trade-off in specificity in some comparisons. Overall, LMF-CP demonstrates favorable predictive performance among the evaluated models on the test set.

### 2.6. Ablation Experiments

To investigate the contribution of different molecular representations and evaluate the benefit of multimodal integration, we conduct ablation experiments by constructing four modality variants of LMF-CP. Each variant retains only one modality while the other three modalities are excluded. A comparative analysis of the LMF-CP model and the four variants above is presented in Table 4. The four variants are defined as follows.

- LMF-CP-MF: It uses only the MACCS fingerprint features as input to predict the carcinogenicity of the compound molecule.
- LMF-CP-MGAT: It applies only the molecular graph feature as the input to the model to predict the carcinogenicity of the compound molecule.
- LMF-CP-MIMA: It employs only the molecular image feature as the input to the model to predict the carcinogenicity of the compound molecule.
- LMF-CP-MSM: It exploits only the SMILES sequence feature as input to predict the carcinogenicity of the compound molecule.

According to Table 4, the AUC, ACC, SEN, SPE, and F1 scores of LMF-CP-MF decrease by 4.4%, 9.1%, 14.1%, 3.9%, and 10.6% compared with LMF-CP. The corresponding decreases for LMF-CP-MGAT are 17.2%, 18.8%, 18.3%, 19.2%, and 18.8%, respectively; those for LMF-CP-MSM are 13.4%, 19.5%, 8.7%, 30.8%, and 16.2%, respectively; and those for LMF-CP-MIMA are 28.1%, 27.7%, 26.4%, 29.1%, and 28.1%, respectively. These results indicate that MACCS fingerprints provide the strongest standalone predictive signal among the four molecular representations, while the complete multimodal framework benefits from the complementary information provided by the additional molecular graph, SMILES, and image modalities.

**Figure 3:**
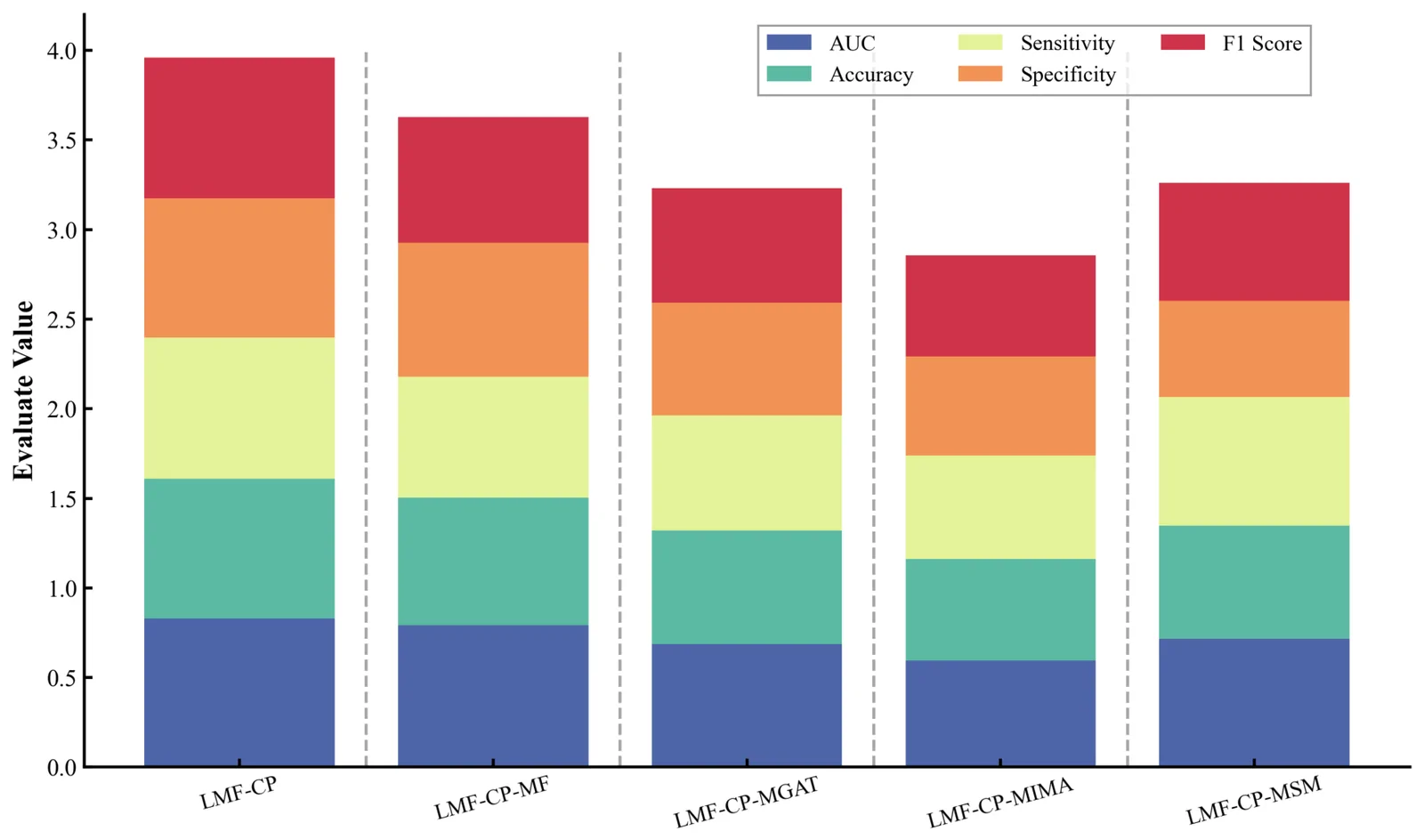
Bar chart comparing performance metrics of various models in ablation experiments. Note: MF = Molecular Fingerprint, MGAT = Molecular Graph, MIMA = Molecular Image, MSM = SMILES Sequence.

To provide an intuitive visualization of performance differences across the five evaluation metrics, we have plotted a bar chart comparison as shown in Figure 3. The ablation results show that no single modality matches the performance of the complete LMF-CP model. This supports the complementary role of molecular fingerprints, molecular graphs, SMILES sequences, and molecular images in the final prediction.

### 2.7. Multimodal Interpretability Analysis of Contributions Based on SHAP

**Figure 4:**
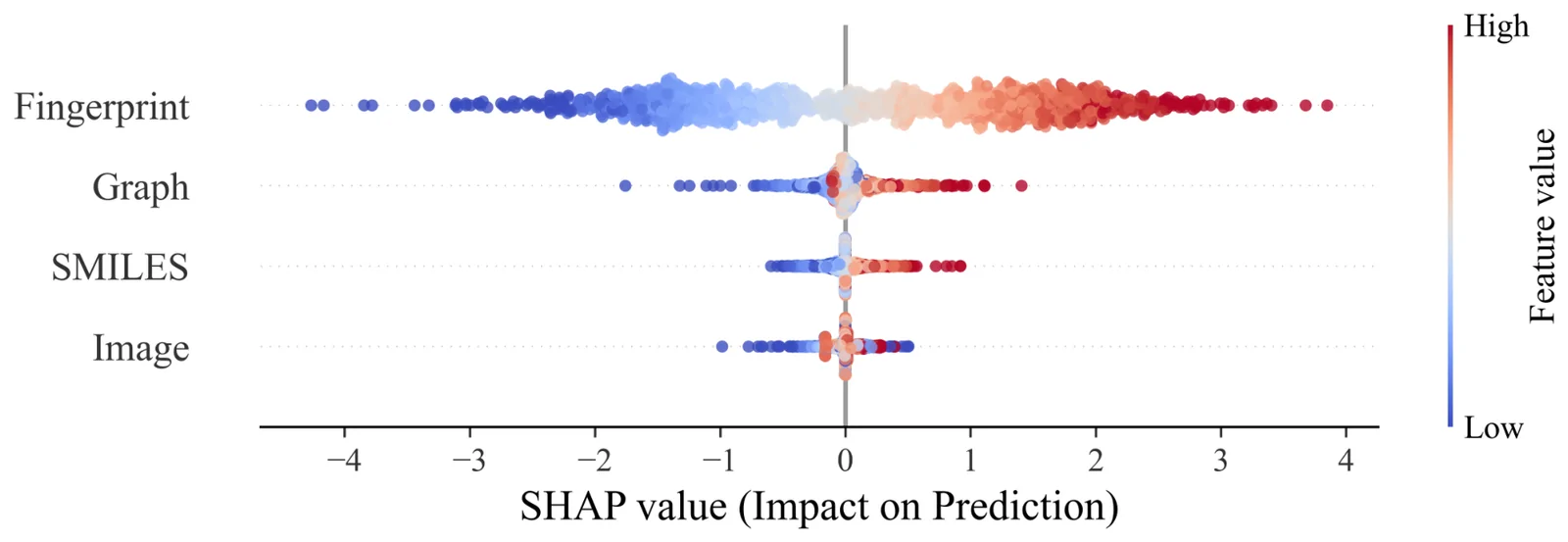
Multimodal SHAP Swarm Plot. Each row in the figure represents a modality, and each point represents a sample.

To examine how different molecular modalities contribute to the final predictions of LMF-CP, this study employs the SHAP method to interpret the outputs of the multimodal fusion model. As a unified interpretive framework based on game theory, SHAP values quantify the marginal contribution of each feature to the output of the model [44]. It should be noted that SHAP characterizes the contribution of input features to model predictions rather than establishing causal relationships between molecular features and carcinogenicity [45,46,47]. Figure 4 displays the SHAP swarm plot, which reveals several key features regarding the contribution of each modality to the predictions of the model. Each row in Figure 4 represents a modality, and each point represents a sample. As we can see, the SHAP value distribution for the molecular fingerprint modality has the widest range, indicating that the prediction output of the molecular fingerprint branch has the largest variation in its contribution to the final classification. In contrast, the SHAP value distributions for the molecular graph and SMILES modalities are more concentrated. While molecular fingerprints capture the primary substructure features, the molecular graphs provide topological information, the SMILES sequences contain contextual information, and the image modality provides additional visual structural information. Overall, molecular fingerprints show the largest variation in SHAP contributions, while the graph, image, and SMILES modalities provide additional complementary information to the final prediction.

**Figure 5:**
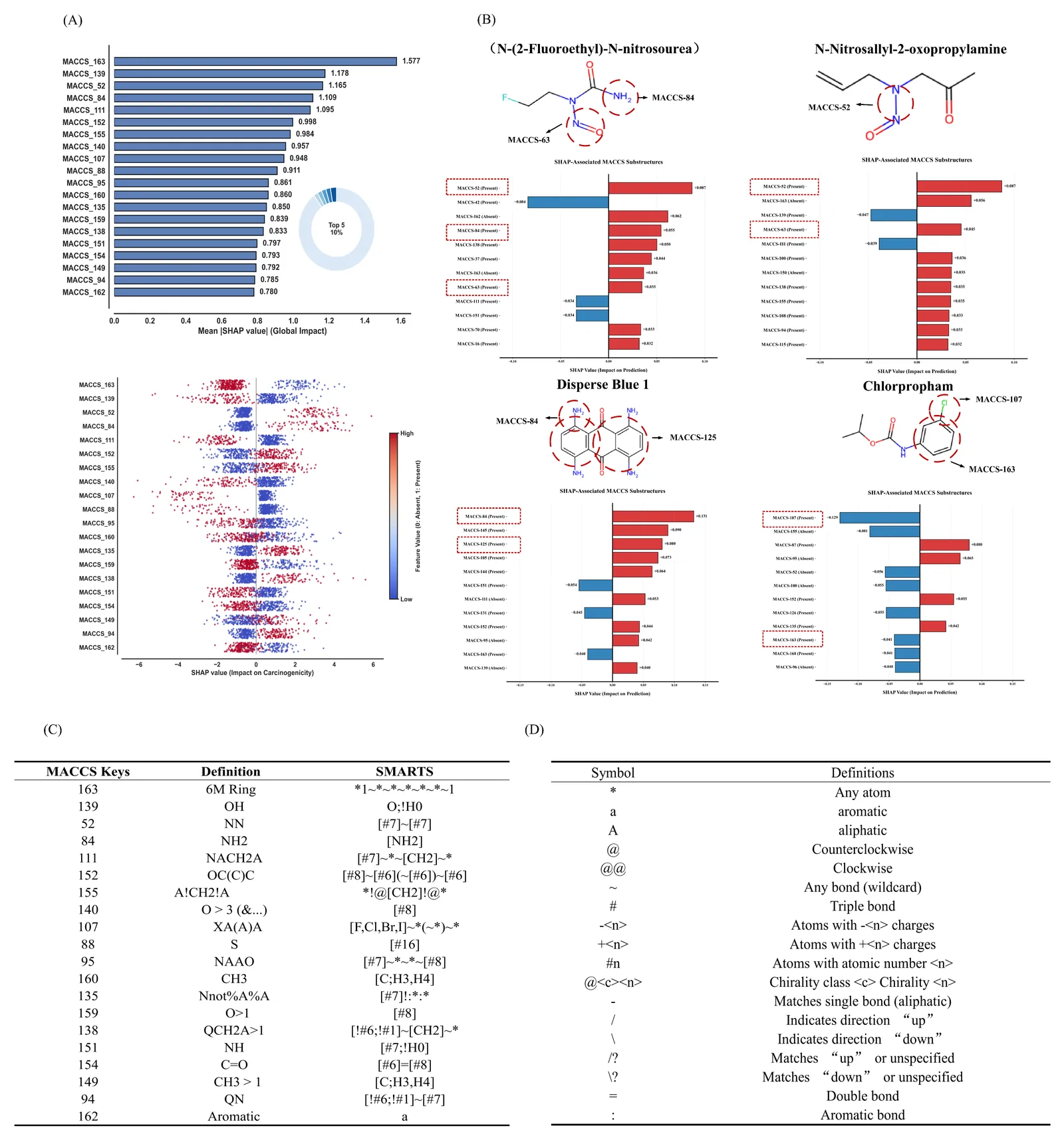
Feature importance analysis for MACCS fingerprints. (**A**) Top 20 MACCS features with the greatest impact on carcinogenicity predictions, ranked by their mean absolute SHAP values. (**B**) Local SHAP explanations for representative compounds, showing the contributions of individual MACCS features to the model predictions; selected MACCS substructures are highlighted with red dashed circles. (**C**) MACCS keys, definitions, SMARTS presentations, and groups of features. (**D**) Definitions of the SMARTS pattern.

Although this study employs multimodal fusion, the highly abstract nature of image and SMILES sequence features makes it difficult to map them directly to specific chemical functional groups. As MACCS fingerprints have clearly defined substructures, we employ the SHAP method to conduct a feature importance analysis of MACCS fingerprints, identifying the key structural features associated with the carcinogenicity predictions of the model. Figure 5A shows the top 20 most important features ranked by their average absolute SHAP values. Based on the chemical structural features represented by these keys, they can be categorized into the following five groups: (1) nitrogen-containing groups, (2) oxygen-containing groups, (3) cyclic structures, (4) halogen-containing groups, and (5) other features (including sulfur-containing and alkyl structures) [48].

Among these, nitrogen-containing groups constitute the largest category. Taking N-(2-fluoroethyl)-N-nitrosourea and N-nitrosallyl-2-oxopropanamine as representative examples, MACCS-52 (N-N bond), MACCS-63 (N=O bond), and MACCS-84 (${\mathrm{NH}}_{2}$) show positive contributions to the corresponding model predictions (Figure 5B).

Next, taking the typical dye Disperse Blue 1 as an example (Figure 5B), this compound is predicted with a high probability of carcinogenicity by the model. Its local SHAP profile shows that MACCS-125 (aromatic ring > 1), MACCS-145 (more than one six-membered ring), and MACCS-84 (${\mathrm{NH}}_{2}$) contribute positively to the model prediction. In contrast, the herbicide chlorpropham is predicted as a non-carcinogen with a low predicted probability. Although this molecule also contains aromatic structural elements, the selected ring-related MACCS features do not exhibit comparably strong positive SHAP contributions.

To further examine the relationship between these structural features and the model outputs, compounds are divided into a higher-prediction group (p>0.7) and a lower-prediction group (p<0.3). As shown in Table 5, the N-N bond occurs in 31.3% of the compounds in the higher-prediction group, compared with 11.6% in the lower-prediction group. Similarly, the N=O bond occurs in 35.2% and 14.4% of the two groups, respectively. The ${\mathrm{NH}}_{2}$ feature and the presence of more than one aromatic ring also occur more frequently in the higher-prediction group. These results indicate that the selected MACCS structural features are more frequently observed among compounds with higher predicted carcinogenicity probabilities.

As several aromatic structural characteristics cannot be fully captured by considering individual MACCS features alone, this study further conducts a Bemis–Murcko scaffold analysis to examine the distribution patterns of core molecular frameworks in the overall dataset. This analysis complements the substructure-level SHAP interpretation by providing a scaffold-level view of structural patterns associated with carcinogenicity.

**Figure 6:**
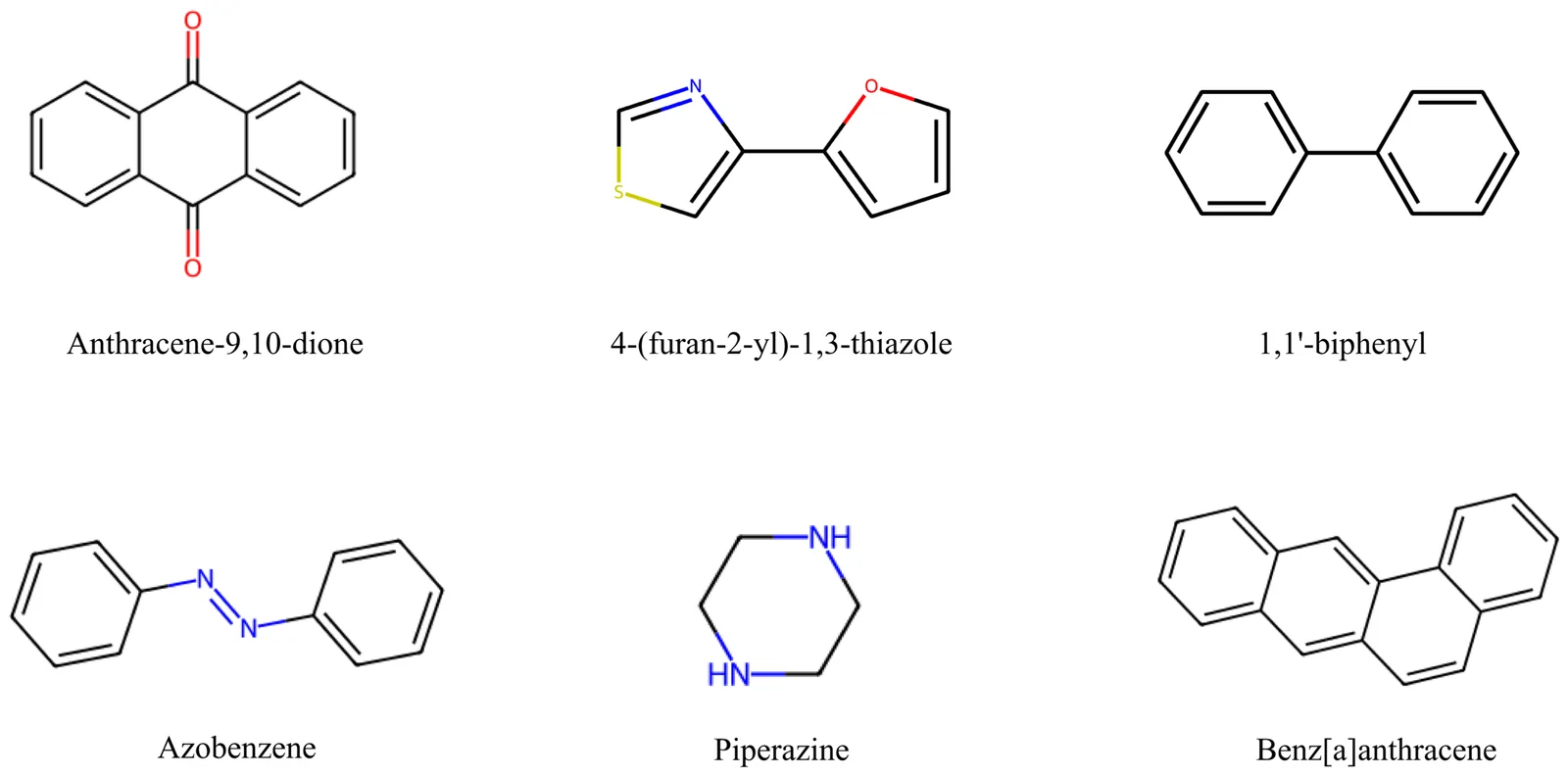
Representative Bemis–Murcko scaffolds enriched in carcinogenic compounds.

To quantitatively evaluate scaffold enrichment, the occurrence of each recurrent Bemis–Murcko scaffold in carcinogenic and non-carcinogenic compounds is compared. For each scaffold, enrichment ratio and odds ratio (OR) are calculated, and differences in scaffold distribution between the two classes are assessed using two-sided Fisher’s exact tests. Detailed scaffold-level enrichment statistics are summarized in Table 6. Figure 6 presents the representative scaffold structures that are enriched in carcinogenic compounds, which primarily include fused polycyclic aromatic structures (e.g., benz[a]anthracene and anthracene-9,10-dione), heterocyclic aromatic systems, simple aromatic scaffolds, and non-aromatic heterocyclic structures. Overall, the scaffold-level analysis complements the preceding MACCS-based SHAP analysis. While the SHAP analysis primarily identifies local substructural features contributing to model predictions, the Bemis–Murcko analysis provides additional information on recurring core molecular frameworks associated with carcinogenic compounds [49,50,51].

### 2.8. LMF-CP Carcinogenicity Prediction Platform

To enhance the usability and practical value of the model, this paper draws on the work of Huynh et al. [27] to further develop a platform for predicting the carcinogenicity of compounds based on multimodal deep learning. This platform is packaged as a user-friendly interactive web application, enabling rapid prediction and visual analysis of compound carcinogenicity. It is publicly available at https://lmfcp-app-hxxzm3u8kvvqb5ephv4q9f.streamlit.app/ (accessed on 2 July 2026).

**Figure 7:**
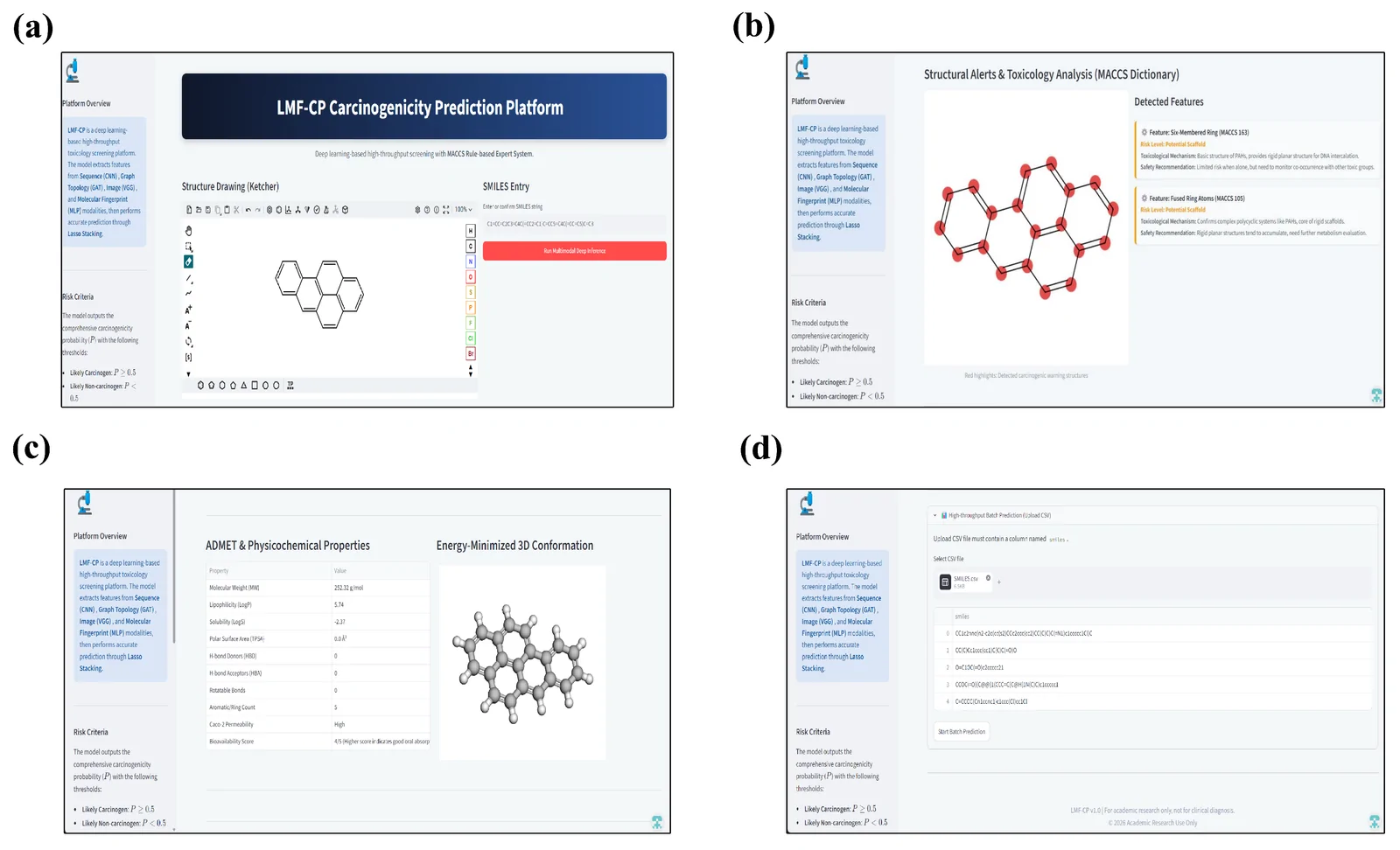
Schematic diagram of an interactive web application for predicting the carcinogenicity of compounds. (**a**) Shows the molecular input interface in single-molecule mode; (**b**) displays selected SHAP-associated MACCS substructures used for structural interpretation; (**c**) shows the physicochemical properties and 3D structure of the molecule; (**d**) is the batch prediction mode, which allows users to upload CSV files containing SMILES strings.

At the application deployment level, this platform utilizes the lightweight Streamlit web framework to build a graphical user interface that supports both single-molecule prediction and batch prediction modes. Figure 7 presents relevant details. The single-molecule prediction mode allows users to directly input the SMILES string of a single compound. The platform returns the predicted carcinogenicity probability and classification result, together with molecular structure visualization and selected physicochemical properties. In the single-molecule prediction mode, the platform primarily offers three functions.

- Enter data. The system prioritizes user-entered SMILES strings and performs standardization checks and atom count verification on them.
- Carcinogenicity prediction. The platform uses the multimodal fusion model to generate probability estimates for the carcinogenicity of compounds and outputs the corresponding prediction results.
- Molecular structure visualization. The platform features built-in fingerprint tracing. For an input compound, the system automatically searches for and highlights selected SHAP-associated MACCS substructures present in the molecule. Furthermore, the platform supports 3D visualization and simultaneously outputs physicochemical property parameters such as LogP, TPSA, and MW to assist researchers in conducting comprehensive evaluations of compounds from both structural and property perspectives.

In addition to single-molecule prediction, the platform also supports batch prediction mode. In this mode, users can upload files containing the SMILES column, and the system automatically performs batch predictions, generating a result file containing prediction probabilities and categories for users to download. The platform also integrates a template download feature to help users standardize input formats, enabling them to complete single molecule predictions in a short amount of time and effectively meeting the practical needs of high-throughput compound screening. It is suitable for scenarios such as compound library screening, early-stage drug toxicology assessment, and environmental contaminant screening.

In summary, this platform provides an efficient and user-friendly computational tool for the rapid assessment of compound carcinogenicity by leveraging the complementary integration of multimodal information and advanced deep learning architectures, offering promising applications in early-stage toxicity screening for drug development and chemical safety assessment.

## 3. Discussion

Accurately identifying the carcinogenicity of compounds is of great significance for drug discovery, chemical safety assessment, and the early screening of potential carcinogens. To address the issues of high cost, long duration, and low throughput associated with traditional experimental methods, as well as the inadequate structural characterization of single-modal computational methods, this study proposes a multimodal fusion carcinogenicity prediction framework, LMF-CP. This framework integrates four types of molecular representations, namely molecular fingerprints, molecular graphs, molecular images, and SMILES strings, to predict and analyze the carcinogenicity of compounds. The experimental results show that LMF-CP achieves favorable overall predictive performance among the evaluated models, particularly in terms of AUC, ACC, and F1 score. However, its comparative advantage varies across individual evaluation metrics and baseline models.

In terms of model architecture, we design corresponding feature extractors for different modalities: MGAT is used for structural topology modeling in the molecular graph modality, VGGNet is used to extract 2D visual features in the molecular image modality, TextCNN is used to extract sequence information in the SMILES modality, and MLP is used to learn molecular substructure information in the molecular fingerprints modality. Building on this foundation, this paper further compares four fusion strategies, including simple concatenation, attention-weighted fusion, SGD stacking, and Lasso stacking. Lasso stacking achieves the highest mean fivefold cross-validation AUC of 0.8405 and ACC of 0.7738 among the evaluated fusion strategies, although the paired differences are not statistically significant. On the test set, the final LMF-CP model achieves an AUC of 0.828, an ACC of 0.782, an F1 score of 0.786, a SEN of 0.786, and a SPE of 0.779. These results indicate that LMF-CP achieves better performance in identifying carcinogenic compounds. Together with the ablation results, these findings support the use of multimodal decision-level fusion for integrating complementary predictive information from different molecular representations.

Regarding interpretability, this study utilizes SHAP-based feature importance analysis to identify structural features associated with carcinogenicity predictions, primarily including nitrogen-containing, oxygen-containing, and ring-related MACCS features. Furthermore, Bemis–Murcko scaffold analysis indicates that the core scaffolds enriched in carcinogenic samples primarily consist of fused aromatic scaffolds, heterocyclic aromatic systems, and certain non-aromatic heterocyclic structures. Together, the SHAP and scaffold analyses provide complementary substructure- and scaffold-level perspectives for interpreting the model predictions.

The structural characteristics identified by the SHAP analysis are generally consistent with previous toxicological evidence. In particular, several nitrogen-containing structural patterns identified in this study are related to toxicologically relevant motifs previously reported in mutagenicity studies [48,49]. Similarly, the positive contribution of aromatic ring features is consistent with the established toxicological relevance of more specific polycyclic aromatic structures [50,51,52]. These consistencies provide additional support for the chemical interpretability of the structural features identified by the model. Nevertheless, SHAP values describe the importance and contribution of individual features to model predictions rather than direct experimental validation of causal carcinogenic mechanisms.

Although this study achieves satisfactory predictive results, certain limitations remain. First, the findings obtained in this study are essentially still structural-level analyses based on computational models. While they can serve as a basis for early warning and auxiliary screening, they cannot directly replace experimental toxicological conclusions and still require further validation through in vitro experiments [53,54]. Second, the current model primarily relies on two-dimensional structural features for prediction and has not yet fully incorporated higher-level information such as three-dimensional conformations [55]. This may limit its ability to capture spatial characteristics such as molecular geometry, steric accessibility, and conformational flexibility. Third, the test set is derived from the current dataset rather than from a completely independent external database. Therefore, the robustness and generalizability of LMF-CP across broader chemical spaces and different databases remain to be further established through independent cross-database validation. Finally, different compounds may involve distinct carcinogenic mechanisms. Therefore, the applicability of the current interpretable results across different chemical classes remains to be further validated [56].

Future research can further expand the datasets and integrate additional molecular representations, particularly three-dimensional conformational information. Specifically, 3D conformations could be generated and optimized using RDKit and molecular force fields, from which geometric information such as bond lengths, bond angles, and interatomic distances could be extracted and encoded using a geometry-aware graph neural network to obtain a 3D molecular representation as an additional modality. Independent database validation will also be important for further assessing the generalizability of the framework. In addition, further efforts can continue to refine the platform deployment and interactive capabilities to advance the practical application of this model in drug discovery and chemical safety assessment. Overall, the results of this study suggest that LMF-CP provides a promising multimodal and interpretable computational framework for preliminary rodent carcinogenicity screening.

## 4. Materials and Methods

### 4.1. Data Preparation

In this paper, the datasets are chosen from three online rodent carcinogenicity databases to develop and better validate our model, and detailed descriptions of each dataset are provided as follows:

- The Carcinogenicity Potency Database (CPDB) is a database of carcinogenicity data based on experiments in mice, rats, and hamsters, containing results from chronic and long-term animal cancer tests conducted since the 1950s [57]. The CPDB data used in this study were obtained from the research by Zhang et al. [20] and included 494 carcinogenic chemicals and 509 non-carcinogenic chemicals.
- The Chemical Carcinogenesis Research Information System (CCRIS) database provides carcinogenicity data for more than 4500 compounds based on rodent studies, containing chemical records of results from carcinogenicity, mutagenicity, tumor promotion, and tumor suppression tests https://www.nlm.nih.gov/databases/download/ccris.html (accessed on 12 December 2025) [58].
- The International Standard Substances for Cancer Bioassays Nomenclature (ISSCAN) database, managed by the National Institute of Public Health, contains results from long-term rodent carcinogenicity studies involving 1150 compounds, which have been reviewed by experts in carcinogenesis and mutagenesis [59].

For the compounds in the datasets described above, data cleaning is performed using the RDKit library [60,61,62], which mainly involves removing compounds containing large amounts of heavy metals, compounds containing fewer than three carbon atoms and mixtures.

Since the compounds in the original datasets are represented only by Chemical Abstracts Service (CAS) Registry Numbers, they must be converted into the format suitable for extracting molecular features. We utilize PubChem to obtain the SMILES [63] for each compound and assign labels to them. After data cleaning and deduplication, 1003 compounds are selected from the CPDB (including 494 carcinogens and 509 non-carcinogens), 887 compounds from the CCRIS database (including 460 carcinogens and 427 non-carcinogens), and 40 compounds from the ISSCAN database (including 23 carcinogens and 17 non-carcinogens). The final dataset contains 1930 unique compounds, including 977 carcinogenic and 953 non-carcinogenic samples. The complete dataset is randomly split into the training set, the validation set, and the independent test set in a 7:2:1 ratio. The test set is kept completely separate from model training, hyperparameter optimization, fusion-strategy comparison, and model selection, and is used only for the final performance evaluation. Finally, the five-fold cross-validation is performed on the training set. In each iteration, four folds are used for model training, while the remaining fold is used for validation. This procedure is repeated five times until each fold has been used once as the validation fold.

**Figure 8:**
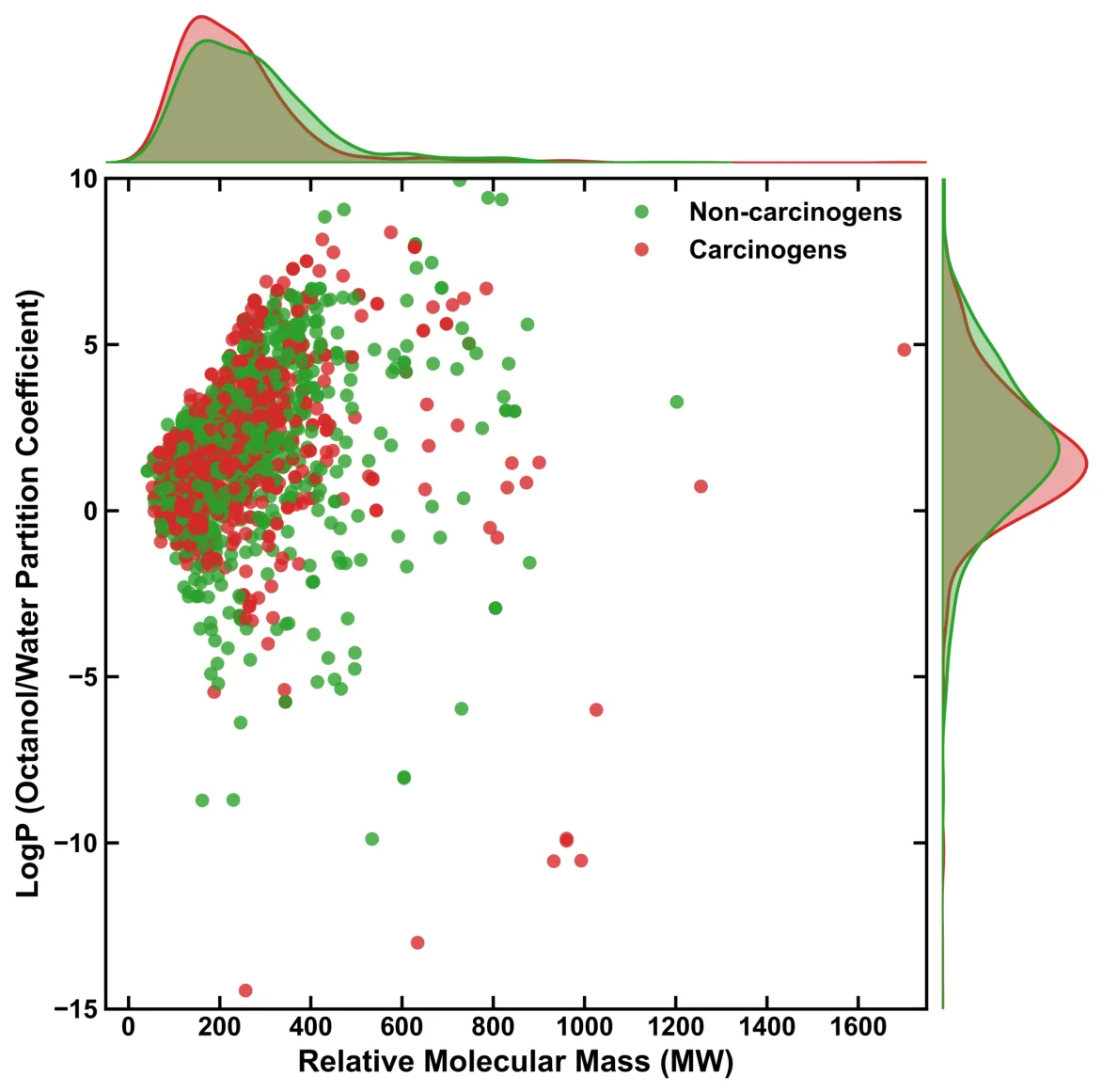
A plot of the chemical space distribution of the training set.

To ensure the reliability of the model, a scatter plot of molecular weight (MW) and partition coefficient (LogP) illustrates the distribution of the chemical space in the training set, as shown in Figure 8 [20]. The results indicate that carcinogenic and non-carcinogenic compounds exhibit similar distribution characteristics, with MW ranging primarily between 50 and 600 Da, and LogP ranging mainly between −5 and 8. This distribution indicates that the training set in this study consists primarily of typical small molecule compounds, and the model is specifically designed for this class of compounds.

### 4.2. Architecture of LMF-CP

**Figure 9:**
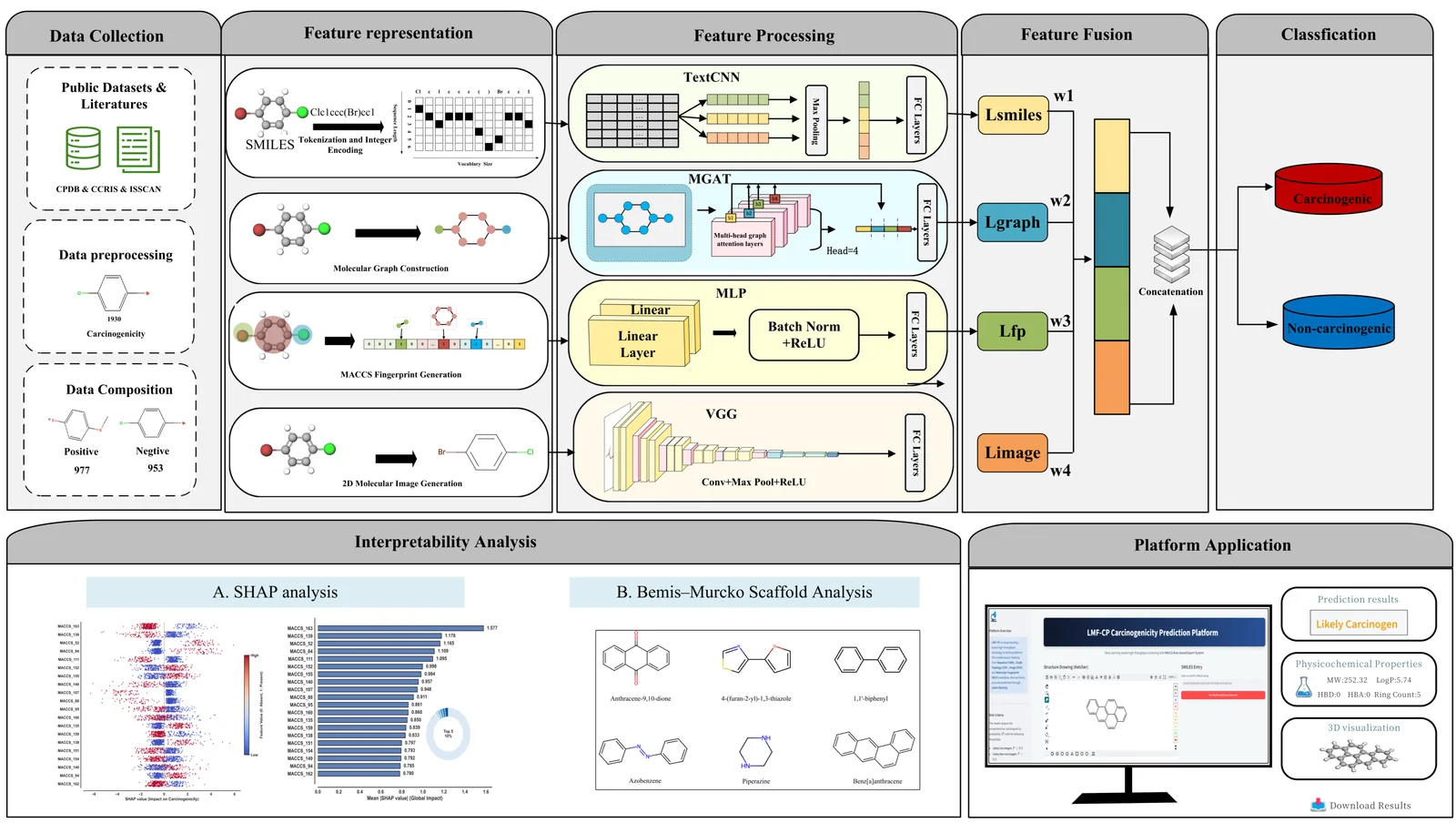
The overall framework of the LMF-CP (Late Multimodal Fusion of Carcinogenicity Prediction).

The overall framework of the LMF-CP model proposed in this study is shown in Figure 9, which primarily includes data collection, multimodal feature representation, feature processing, feature fusion, classification, interpretability analysis, and platform applications. First, during the data collection stage, positive and negative carcinogenicity samples are primarily sourced from public datasets and relevant literature. Second, in the multimodal feature extraction stage, feature extraction networks are constructed independently to process four different representations of the compounds. Molecular fingerprints are mapped using the MLP, topological structure information is extracted from molecular graphs using the MGAT, visual features are extracted from two-dimensional molecular images using the VGGNet, and sequence information is extracted from SMILES sequences using the TextCNN. Then, in the multimodal fusion stage, the modality logits from each unimodal base learner are extracted as secondary features and fed into the decision layer. To effectively utilize information from different modalities, we compare several ensemble strategies, consisting of simple concatenation, attention-weighted fusion, stacking based on stochastic gradient descent (SGD), and the Lasso algorithm. For model interpretation, SHAP analysis is first used to characterize the contributions of different molecular modalities to the model output and is then further applied to MACCS fingerprints to identify prediction-associated substructural patterns. Additionally, Bemis–Murcko scaffold analysis is conducted to investigate representative core molecular frameworks associated with carcinogenic compounds, complementing the substructure-level analysis. Finally, this study develops a visual online prediction platform (https://lmfcp-app-hxxzm3u8kvvqb5ephv4q9f.streamlit.app/ (accessed on 2 July 2026)), which supports single-molecule and batch predictions, facilitating the rapid assessment of the carcinogenic risk of the compound.

### 4.3. Feature Representation

**Figure 10:**
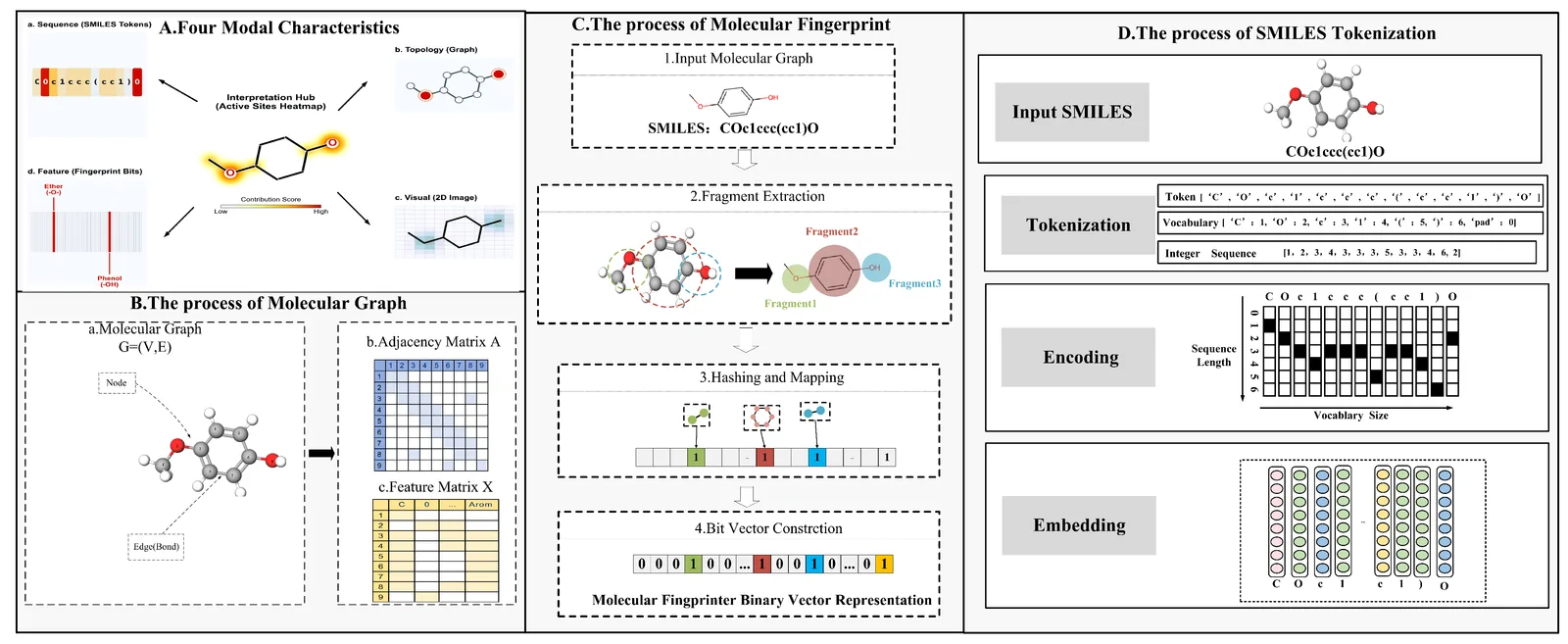
Molecular representations of molecules. Molecules can be described using SMILES-encoded vectors, molecular fingerprints, molecular images, and molecular graph.

To convert complex molecular data into molecular structures and chemical information that can be recognized and learned by the LMF-CP model, feature extraction is first performed on the molecules. During this process, four types of features are extracted for each molecule: SMILES sequences, molecular graph features, molecular fingerprint features, and molecular images. The specific extraction process is shown in Figure 10.

#### 4.3.1. Molecular Fingerprints

MACCS fingerprints [64] are a molecular characterization method based on chemical substructures, which converts complex chemical molecules into a binary vector consisting of 0 and 1 by checking for the presence of specific chemical structural features within the molecule [65]. These structural features play a crucial role in explaining molecular activity, and this coding method is known as “fingerprint encoding”. A specific schematic diagram is shown in Figure 10C. In drug discovery, the MACCS fingerprints are commonly used to screen compound libraries for potentially active molecules. Software tools for calculating fingerprints include CDK [66], RDKit (Version 2024.03.6) [67] and Padel-Descriptor software (Version 2.21) [68]. Currently, molecular fingerprints are employed to represent molecules in many compound toxicity prediction models.

#### 4.3.2. Molecular Graph Feature

The features of the molecular graph consist mainly of two matrices, as shown in Figure 10B, where $A_{N\times N}$ is the adjacency matrix and $X_{N\times F}$ is the node feature matrix, N is the number of atomic nodes, F is the number of atomic node features [69]. The node feature matrix X includes atom types, number of degrees, number of bound hydrogens, implicit valence, size of the ring containing the atom, and aromaticity, which are shown in Table 7. These atom descriptors are converted to a one-hot encoded feature vector.

#### 4.3.3. SMILES Tokenization and Vectorization

The SMILES string is a chemical notation that describes chemical structures using ASCII characters, and is considered a low-level representation of molecular structures [70]. To convert the SMILES string into the format that the model can process, tokenization and vectorization processes are applied. For example, the SMILES string “CS(=O)(=O)Cl” can be decomposed into the sequence [“C”, “S”, “(”, “=”, “O”, “)”, “(”, “=”, “O”, “)”, “Cl”] [38,71]. The specific processing steps are shown in Figure 10D, which mainly involve the following steps.

- Tokenization. Split a continuous SMILES string into the smallest units (tokens), each of which possesses independent chemical semantics.
- Build Vocabulary. Iterate through the entire dataset and assign a unique integer index to each distinct token. At the same time, special placeholders (such as “<PAD>”) are introduced into the vocabulary by padding and aligning the molecular sequences so that the sequences can be uniformed the same lengths.
- Embedding. Discrete integer sequences are converted into vectors that can be processed by the model.

#### 4.3.4. Molecular Image Feature

In this paper, SMILES sequences are converted into PNG images of 224 × 224 pixels using the Draw.MolToImage in the RDKit library [72].

### 4.4. Feature Processing Stage

To enhance the expressive power of each molecular feature, we first select substructure fingerprints (MACCS fingerprints) as molecular fingerprints. Second, for molecular graph features, atomic, bond, and topological information are captured by MGAT. Then, SMILES sequences are tokenized and vectorized by TextCNN. Finally, molecular images are processed using VGGNet. The detailed methods and steps for processing these molecular features are listed in Figure 9.

#### 4.4.1. Molecular Graph Attention Network

Molecular Graph Attention Network (MGAT) combines a graph neural network and an attention mechanism, in which the structural features are learned and characterized effectively [73]. For solving graph structure problems, unlike traditional methods, MGAT introduces an attention mechanism and allows each node to assign different weights to its neighbors, which can better capture the dependencies between nodes.

The input variables to MGAT are a set of atomic node features. Given a set of atomic node features $s=\left\{s_{1},s_{2},\ldots ,s_{N}\right\}\left(s_{i}\in R^{F},i=1,2,\ldots ,N\right)$, where N is the number of atomic nodes, $R^{F}$ is a set comprising all atomic node features and F is the number of atomic node features. Each layer generates a new set of atomic node feature vectors $s^{\prime }=\left\{s_{1}^{\prime },s_{2}^{\prime },\ldots ,s_{N}^{\prime }\right\}\left(s_{i}^{\prime }\in R^{F^{\prime }},i=1,2,\ldots ,N\right)$ as output. Here, $R^{F^{\prime }}$ is the set composed of these new atomic node features, and $F^{\prime }$ denotes the dimension of the new atomic node feature vectors.

Step 1: Feature projection and compute attention coefficients. In order to obtain better expressiveness, we employ multi-layer stacking and linear transformations. First, we apply a weight matrix $W\in R^{F^{\prime }\times F}$ for each atomic node to share linear transformations. Then, we use a shared attention mechanism on each atomic node to compute attention coefficients, and the formula for the attention coefficient is as follows:

(5) $$e_{ij}=\delta \left(Ws_{i},Ws_{j}\right),$$

where $e_{ij}$ represents the feature importance of atomic node j to atomic node i, and δ is an attention function used to calculate the correlation between two transformed features.

Step 2: Softmax normalization. To ensure more accurate weight assignment, the normalization is performed by using the Softmax function to achieve a more balanced and efficient weighting scheme, which can be calculated by the following equations:

(6) $$\alpha_{ij}=Softmax\left(e_{ij}\right)=\frac{\exp(e_{ij})}{\sum_{k\in N_{i}}\exp\left(e_{ik}\right)},$$

where $\alpha_{ij}$ denotes the normalized attention coefficient from node j to node i. Then we apply the LeakyReLU function to compute $e_{ij}$ and $e_{ik}$ as follows:

(7) $$\begin{array}{cc} & \alpha_{ij}=\frac{\exp\left(\mathrm{LeakyReLU}\left(\delta^{T}\left[Ws_{i}∥Ws_{j}\right]\right)\right)}{\sum_{k\in N_{i}}\exp\left(\mathrm{LeakyReLU}\left(\delta^{T}\left[Ws_{i}∥Ws_{k}\right]\right)\right)} \end{array},$$

where $\delta \in R^{2F^{\prime }}$ is the weight vector in a single-layer forward propagation network, $\delta^{T}$ denotes the transpose of δ, and “||” denotes the concatenation operation. We use Equation (7) to compute the linear combination of the corresponding features as the final output features of each atomic node.

Step 3: Feature aggregation to generate new node representations. Finally, each node updates its representation by aggregating the features of its neighbors; the nonlinear activation function σ is applied, and the final feature vector output $s_{i}^{\prime }$ by node *i* is defined as follows:

(8) $$s_{i}^{\prime }=\sigma \left(\sum_{j\in N_{i}}\alpha_{ij}Ws_{j}\right).$$

Step 4: Multi-head attention mechanism. The single attention head may be overly biased toward a particular neighbor, whereas multiple heads can provide different perspectives to balance this bias. Thus, multi-head attention can be obtained by repeating Step 1–Step 3, and it can be calculated by the following equation:

(9) $$S_{i}^{\prime }={∥}_{k=1}^{m}\sigma \left(\sum_{j\in N_{i}}\alpha_{ij}^{k}W^{k}s_{j}\right),$$

where m denotes the total number of attention heads, $S_{i}^{\prime }$ denotes the new feature representation of node *i* after multiple attention layers, and $\alpha_{ij}^{k}$ denote the attention weight from node *j* to node *i* in the kth attention head. In this paper, the outputs from each MGAT are linked to derive the final feature vector for the molecular graph.

#### 4.4.2. Text Convolutional Neural Network

Text Convolutional Neural Network (TextCNN) is a text classification model based on Convolutional Neural Networks (CNNs) that is widely used in various text classification tasks. Unlike CNNs used in traditional image classification, TextCNN takes the text matrix as input variables rather than images [74,75], and its core idea is to extract important semantic features from text through convolutional operations using convolutional kernels of various sizes. TextCNN, with a simple structure, low computational complexity, and high computational efficiency, has been widely adopted in the field of text classification [71].

TextCNN typically consists of four layers for text classification tasks.

- Input Layer. The input variable is the N×K word vector matrix, where *N* is the number of words and *K* is the dimension of the word embeddings. Each word is mapped to a word vector of the fixed dimension, forming the word vector matrix.
- Convolution Layer. Each convolutional kernel slides across the input matrix at a fixed stride and extracts a local feature at each sliding position, and multiple convolutional kernels can extract different types of feature information.
- Pooling Layer. Pooling methods include max pooling and average pooling, which are used to reduce the complexity of features and parameters, and can effectively prevent overfitting.
- Output Layer. Classification predictions are made using a fully connected layer and the sigmoid function.

In this study, TextCNN is used as the base learner within the multimodal framework, where the input sequence is padded to the fixed length L=109, and each token is mapped to an integer index based on the pre-built dictionary. Thus, the integer index sequences are transformed into embedding vectors via the embedding layer. Three one-dimensional convolutional kernels of different sizes are used to extract multi-scale chemical features in parallel. Finally, the predicted value for the SMILES modality is generated by the classification head.

#### 4.4.3. Visual Geometry Group Network

**Figure 11:**
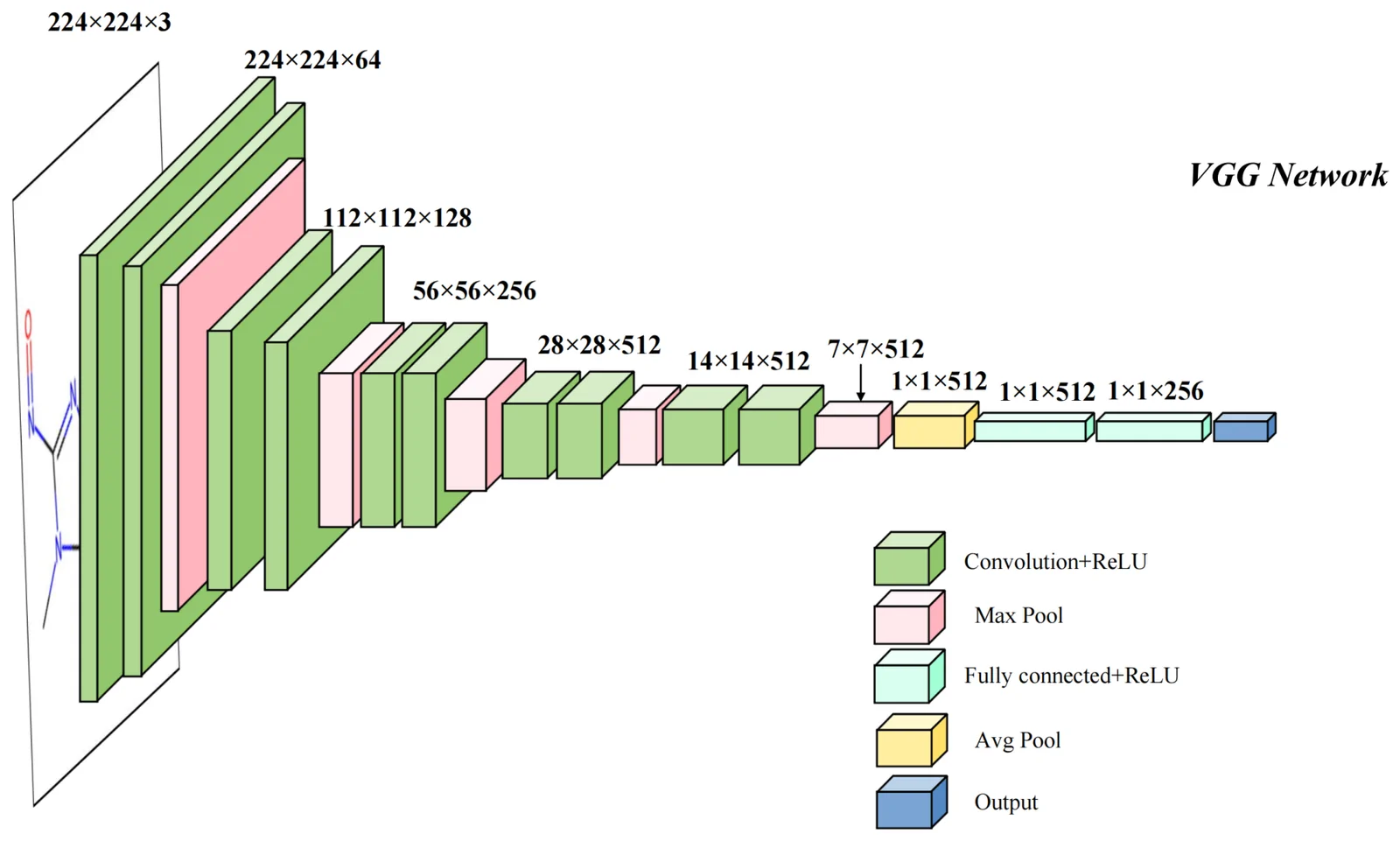
Architecture of the VGGNet used for molecular image feature extraction.

To extract features from molecular images that are relevant to the overall molecular structure and properties, VGGNet as an image encoder is employed to process molecular image features. Compared to shallow CNN models, VGGNet possesses stronger feature learning and representation capabilities. In particular, VGGNet can learn more effective feature information from the data when handling complex image tasks, which can significantly improve recognition performance [76]. In addition, VGGNet requires RGB images of a fixed size of 224 × 224 pixels as input variables, and the VGGNet architecture is shown in Figure 11. The network consists of 10 convolutional layers and 2 fully connected layers, with all convolutional kernels having a size of 3 × 3 in the convolutional layers. The network also includes five max-pooling layers responsible for spatial pooling of the features, and the pooling operates on a 2 × 2 pixel window with a stride of two pixels. In the first two convolutional layers of the VGGNet, each layer contains 64 convolutional kernels; the third and fourth layers each contain 128 kernels; the fifth and sixth layers each contain 256 kernels; the seventh and eighth layers each contain 512 kernels; and the ninth and tenth layers each contain 512 kernels. The final output layer consists of the average pooling layer and the fully connected layer. The output of the convolutional layers is represented as shown in the formula below:

(10) $$y^{\left(l\right)}=\sigma \left(W^{\left(l\right)}⊗X^{\left(l-1\right)}+b^{\left(l\right)}\right),$$

where $y^{\left(l\right)}$ is the output feature vector of the lth convolutional layer, σ is the ReLU activation function, $W^{\left(l\right)}$ and $b^{\left(l\right)}$ are the weights and biases of that layer, respectively.

### 4.5. Feature Fusion Module

Multimodal approaches are widely used in the field of drug discovery. However, situations such as redundant, synergistic, or complementary modal information [77,78] may occur during multimodal fusion. Accordingly, the choice of fusion strategy is important for effectively integrating multimodal information. In this paper, we will provide a brief overview of three feature fusion techniques, consisting of early fusion, mid-stage fusion, and late fusion, each of which offers unique advantages.

**Figure 12:**
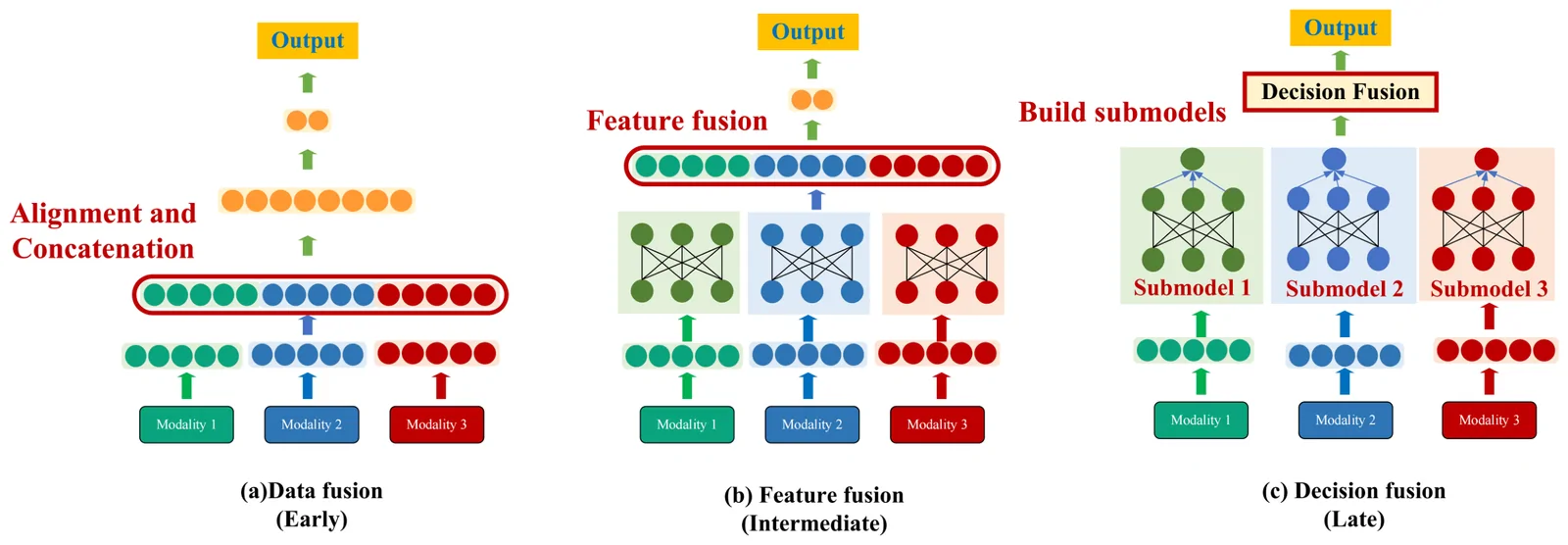
Multimodal information fusion stage: (**a**) shows fusion at the data layer, (**b**) shows fusion at the feature layer, and (**c**) shows fusion at the decision layer.

Data-level fusion, also known as early fusion, is illustrated in Figure 12a. Data-level fusion involves preprocessing and directly concatenating data from different modalities. This method results in minimal information loss and is simple to implement. However, direct concatenation may disrupt the local structure of the data, preventing information from being effectively conveyed.

Feature-level fusion, also known as mid-stage fusion, is shown in Figure 12b. Feature-level fusion extracts features from each modality and fuses them in a unified feature space. The complementarity between modalities can be better leveraged, but the choice of strategy depends on the data and the task.

Decision-level fusion, often referred to as late-stage fusion, is shown in Figure 12c. Based on ensemble learning, this approach trains independent submodels for each modality and fuses their predictions to improve prediction accuracy.

To fully integrate the complementary feature information across different modalities, this paper compares four multimodal fusion strategies. The first strategy involves direct concatenation, where the feature outputs from the encoders of the four modalities are concatenated and fed into a fully connected layer. The second strategy employs feature fusion based on attention, introducing an attention mechanism at the feature level. The third strategy involves learning weights at the decision layer, where each modality outputs its own prediction independently, and then Lasso and SGD are used to optimize the weighted combination, respectively.

## 5. Conclusions

This study proposes LMF-CP, a multimodal fusion framework for carcinogenicity prediction, which integrates four molecular representations, namely molecular fingerprints, molecular graphs, molecular images, and SMILES sequences. Experimental results show that LMF-CP achieves favorable overall predictive performance among the evaluated models, although the magnitude of its comparative advantage varies across individual metrics. On the test set, LMF-CP achieves an AUC of 0.828, an ACC of 0.782, and an F1 score of 0.786. In addition, SHAP-based feature importance analysis and Bemis–Murcko scaffold analysis reveal complementary local substructural features and core molecular scaffolds associated with the model predictions. These analyses improve the interpretability of LMF-CP by providing structural insights into the features and molecular frameworks contributing to its prediction outputs. Overall, LMF-CP provides a promising and interpretable computational framework for the early screening of compound carcinogenicity, while further independent cross-database validation remains necessary to establish its generalizability across broader chemical spaces.

## Figures and Tables

**Table 1:** Performance comparison of fusion strategies.

Fusion Strategy	ACC	AUC	SEN	SPE
Simple concatenation	0.7720 ± 0.0311	0.8388 ± 0.0128	0.7941 ± 0.0375	0.7494 ± 0.0400
Attention-weighted fusion	0.7692 ± 0.0224	0.8382 ± 0.0161	**0.7964 ± 0.0318**	0.7412 ± 0.0284
SGD stacking	0.7219 ± 0.0950	0.8170 ± 0.0537	0.6770 ± 0.2953	**0.7682 ± 0.1142**
Lasso stacking	**0.7738 ± 0.0281**	**0.8405 ± 0.0154**	0.7896 ± 0.0172	0.7576 ± 0.0440

Notes: ACC: Accuracy, AUC: Area Under Curve, SEN: Sensitivity, SPE: Specificity. Values represent mean ± standard deviation from 5-fold cross-validation. Best results are highlighted in bold.

**Table 2:** Five-fold cross-validation performance of LMF-CP and the baseline models.

Model	ACC	AUC	SEN	SPE
KNN	0.7000±0.0215	0.7596±0.0144	0.7156±0.0325	0.6841±0.0390
SVM	0.7185±0.0240	0.7904±0.0261	0.6826±0.0501	0.7552±0.0289
GCN	0.7242±0.0131	0.7847±0.0217	0.7259±0.0304	0.7225±0.0487
DCAMCP	0.7220±0.0223	0.8056±0.0199	0.8260±0.0417	0.6154±0.0529
LMF-CP	0.7738±0.0281	0.8405±0.0154	0.7896±0.0172	0.7576±0.0440

Note: The best result for each metric is highlighted in bold.

**Table 3:** Performance comparison of LMF-CP with other models on the test set.

Model	AUC	ACC	SEN	SPE	F1
KNN	0.754	0.689	0.663	0.715	0.684
SVM	0.817	0.751	0.694	**0.810**	0.739
GCN	0.792	0.746	0.765	0.726	0.754
DCAMCP	0.808	0.735	0.775	0.694	0.749
**LMF-CP**	**0.828**	**0.782**	**0.786**	0.779	**0.786**

Note: The best result for each metric is highlighted in bold.

**Table 4:** Performance of LMF-CP and single-modality variants on the test set.

Model	AUC	ACC	SEN	SPE	F1
**LMF-CP**	**0.8284**	**0.7824**	**0.7857**	**0.7789**	**0.7857**
LMF-CP-MF	0.7921	0.7110	0.6746	0.7483	0.7023
LMF-CP-MGAT	0.6857	0.6356	0.6417	0.6292	0.6383
LMF-CP-MIMA	0.5958	0.5653	0.5779	0.5523	0.5652
LMF-CP-MSM	0.7176	0.6297	0.7176	0.5389	0.6584

Note: MF = Molecular Fingerprints, MGAT = Molecular Graph, MIMA = Molecular Image, MSM = SMILES Sequence. Best results for each metric are highlighted in bold.

**Table 5:** Distribution of selected MACCS structural features in the higher-prediction and lower-prediction groups.

MACCS Structural Feature	MACCS Key	Higher-Prediction Group	Lower-Prediction Group
${\mathrm{NH}}_{2}$	84	24.0%	19.9%
N-N	52	31.3%	11.6%
N=O	63	35.2%	14.4%
Aromatic ring > 1	125	42.5%	28.8%

**Table 6:** Enrichment statistics of representative Bemis–Murcko scaffolds associated with carcinogenic compounds.

Bemis–Murcko Scaffold	Name	Positive	Negative	*p*	OR	Enrichment Ratio
O=C1c2ccccc2C(=O)c2ccccc21	Anthracene-9,10-dione	18	2	0.00038	8.92	8.78
c1coc(-c2cscn2)c1	4-(furan-2-yl)-1,3-thiazole	14	1	0.00095	13.84	13.65
c1ccc(-c2ccccc2)cc1	1,1′-biphenyl	41	16	0.00112	2.57	2.50
c1ccc(N=Nc2ccccc2)cc1	Azobenzene	7	0	0.01551	Inf	Inf
C1CNCCN1	Piperazine	8	1	0.03876	7.86	7.80
C(=Cc1ccccc1)c1ccccc1	1,2-Diphenylethene	9	2	0.06490	4.42	4.39
c1ccc2c(c1)Cc1ccccc1-2	9,10-Dihydrophenanthrene	11	4	0.11747	2.70	2.68
c1ccc2cc3c(ccc4ccccc43)cc2c1	Benz[a]anthracene	5	1	0.21824	4.89	4.88

Note: Positive and Negative denote the numbers of carcinogenic and non-carcinogenic compounds containing the corresponding scaffold, respectively. OR denotes the odds ratio obtained from Fisher’s exact test; Inf indicates an infinite OR resulting from zero observations in the non-carcinogenic group. *p*-values are from two-sided Fisher’s exact tests.

**Table 7:** Atomic feature descriptors used in molecular graph representation.

Atomic Property	Information	Size
Atomic type	C, N, O, S, F, H, Si, P, Cl, Br,	
	Li, Na, K, Mg, Ca, Fe, As, Al, I, B,	
	V, Tl, Sb, Sn, Ag, Pd, Co, Se, Ti, Zn,	43
	Ge, Cu, Au, Ni, Cd, Mn, Cr, Pt, Hg, Pb,	
	Yb, In, Zr	
Degree	0, 1, 2, 3, 4, 5, 6, 7, 8, 9, 10	11
Number of hydrogen atoms	0, 1, 2, 3, 4, 5, 6, 7, 8, 9, 10	11
Implicit valence	0, 1, 2, 3, 4, 5, 6, 7, 8, 9, 10	11
Size of ring containing the atom	3, 4, 5, 6, 7, 8	6
Aromaticity	True or False	1
Total		83

## Data Availability

The original contributions presented in this study are included in the article. Further inquiries can be directed to the corresponding author. A freely accessible web server for carcinogenicity prediction is available at https://lmfcp-app-hxxzm3u8kvvqb5ephv4q9f.streamlit.app/ (accessed on 2 July 2026).

## Supplementary Materials

The following supporting information can be downloaded at: https://www.mdpi.com/article/10.3390/ijms27167499/s1.
