# Supplementary material for: LMF-CP: An Interpretable Multimodal Late-Fusion Framework for Compound Carcinogenicity Prediction
Source: Int J Mol Sci. 2026 Aug 21;27(16):7499. doi: 10.3390/ijms27167499 (PMC13512965; doi:10.3390/ijms27167499)
Supplement: Supplementary file 1 [file ijms-27-07499-s001.zip › ijms-4457433-supplementary.pdf]

LMF-CP: An Interpretable Multimodal Late-Fusion Framework for Compound Carcinogenicity Prediction

Yingjie Zhu *, Liujie He and Xinjie Liang

School of Mathematics and Statistics, Changchun University, Changchun 130022, China;
19591370365@163.com (L.H.); 135lxjyyds@gmail.com (X.L.)
* Correspondence: zhuyj@ccu.edu.cn (Y.Z.)

Supplementary Information

Table S1. Paired statistical comparisons between Lasso stacking and alternative fusion strategies.

Metric	Comparision	Mean difference	Wins	W_statistic	Raw_p	Holm_p	Rank-biserial
AUC	Lasso VS Simple concatenatior	0.001682	3	5.0	0.6250	0.9375	0.333333
AUC	Lasso VS Attention fusion	0.002291	3	4.0	0.4375	0.9375	0.466667
AUC	Lasso VS SGD stacking	0.023448	4	3.0	0.3125	0.9375	0.600000
ACC	Lasso VS Simple concatenatior	0.001719	2	3.0	0.5000	L.0000	0.400000
ACC	Lasso VS Attention fusion	0.004613	2	4.0	0.8750	L.0000	0.200000
ACC	Lasso VS SGD stacking	0.051873	4	1.0	0.1875	0.5625	0.800000
F1	Lasso VS Simple concatenatior	0.000639	2	4.0	0.8750	1.0000	0.200000
F1	Lasso VS Attention fusion	0.002382	2	7.0	1.0000	1.0000	0.066667
F1	Lasso VS SGD stacking	0.107490	3	3.0	0.3125	0.9375	0.600000
SEN	Lasso VS Simple concatenatior	-0.004545	2	5.0	1.0000	1.0000	0.000000
SEN	Lasso VS Attention fusion	-0.006805	3	6.0	0.8125	1.0000	-0.200000
SEN	Lasso VS SGD stacking	0.112552	3	7.0	1.0000	1.0000	0.066667
SPE	Lasso VS Simple concatenatior	0.008201	3	2.0	0.3750	1.0000	0.600000
SPE	Lasso VS Attention fusion	0.016347	4	4.0	0.4375	1.0000	0.466667
SPE	Lasso VS SGD stacking	-0.010628	3	6.0	0.8125	1.0000	0.200000

Table S2. Paired statistical comparisons between LMF-CP and baseline models.

Metric	Comparision	Mean difference	Wins	W_statistic	Raw_p	Holm_p	Rank-biserial
AUC	LMF-CP VS KNN	0.080869	5	0.0	0.0625	0.250	1.000000
AUC	LMF-CP VS SVM	0.050100	5	0.0	0.0625	0.250	1.000000
AUC	LMF-CP VS GCN	0.055812	5	0.0	0.0625	0.250	1.000000
AUC	LMF-CP VS DCAMCP	0.034838	5	0.0	0.0625	0.250	1.000000
ACC	LMF-CP VS KNN	0.073721	5	0.0	0.0625	0.250	1.000000
ACC	LMF-CP VS SVM	0.055272	5	0.0	0.0625	0.250	1.000000
ACC	LMF-CP VS GCN	0.049543	5	0.0	0.0625	0.250	1.000000
ACC	LMF-CP VS DCAMCP	0.051819	5	0.0	0.0625	0.250	1.000000
F1	LMF-CP VS KNN	0.072598	5	0.0	0.0625	0.250	1.000000
F1	LMF-CP VS SVM	0.069768	5	0.0	0.0625	0.250	1.000000
F1	LMF-CP VS GCN	0.052588	5	0.0	0.0625	0.250	1.000000
F1	LMF-CP VS DCAMCP	0.029337	5	0.0	0.0625	0.250	1.000000
SEN	LMF-CP VS KNN	0.073981	5	0.0	0.0625	0.250	1.000000
SEN	LMF-CP VS SVM	0.106922	5	0.0	0.0625	0.250	1.000000
SEN	LMF-CP VS GCN	0.063701	5	0.0	0.0625	0.250	1.000000
SEN	LMF-CP VS DCAMCP	-0.03641	1	2.0	0.1875	0.250	-0.733333
SPE	LMF-CP VS KNN	0.073487	4	1.0	0.1250	0.375	0.866667
SPE	LMF-CP VS SVM	0.002407	2	7.0	1.0000	1.000	-0.066667
SPE	LMF-CP VS GCN	0.035081	3	4.0	0.4375	0.875	0.466667
SPE	LMF-CP VS DCAMCP	0.142228	5	0.0	0.0625	0.250	1.000000

Table S3. Comparison with recent pretrained molecular representation models

Model	ACC	AUC	F1	SEN	SPE
Mole-BERT	0.6978 ± 0.0163	0.7669 ± 0.0292	0.6791 ± 0.0206	0.6325 ± 0.0309	0.7645 ± 0.0243
ChemBERTa-2	0.6874 ± 0.0294	0.7408 ± 0.0473	0.6870 ± 0.0365	0.6804 ± 0.0562	0.6947 ± 0.0253
**LMF-CP**	**0.7738 ± 0.0281**	**0.8405 ± 0.0154**	**0.7796 ± 0.0245**	**0.7896 ± 0.0172**	**0.7576 ± 0.0440**

Table S4. Performance of LMF-CP under randomized-label negative control experiments.

Setting	Permutation seed	AUC	ACC	SEN	SPE
**True labels**	**-**	**0.8405 ± 0.0154**	**0.7738 ± 0.0281**	**0.7896 ± 0.0172**	**0.7576 ± 0.0440**
Randomized labels	42	0.4904± 0.0359	0.4963± 0.0199	0.3122± 0.2871	0.6855± 0.2818
Randomized labels	66	0.5671± 0.0131	0.5388± 0.0212	0.49946± 0.1409	0.5839± 0..1139
Randomized labels	2026	0.4928± 0.0198	0.4934± 0.0039	0.2728± 0.3355	0.7188± 0.3400
